# Valorization of coffee silver skin and almond skin as a source of dietary fiber, phenolics, and protein in muffins

**DOI:** 10.1016/j.fochx.2026.104755

**Published:** 2026-11-04

**Authors:** Tanveer Ahmad, Francesco Esposito, Muhammad Arham Rauf, Mian Kamran Sharif, Bridget Atubukha, Katalin Badak-Kerti, Salvatore Velotto, Szilvia Banvolgyi, Teresa Cirillo

**Affiliations:** a Department of Agricultural Sciences, University of Naples Federico II, Piazza Carlo di Borbone, 1 - Portici (NA), Italy; b National Institute of Food Science and Technology, University of Agriculture, Faisalabad 38000, Pakistan; c Institute of Food Science and Technology, Hungarian University of Agriculture and Life Sciences, Budapest, Hungary.; d Department of Promotion of Human Sciences and the Quality of Life, University of Study of Roma “San Raffaele”, Roma, Italy

**Keywords:** Functional food, Bakery products, Coffee silver skin, Almond skin, Muffins, Dietary fiber

## Abstract

Agro-industrial by-products are gaining prominence as valuable resources in global initiatives to mitigate food waste and support the transition towards a sustainable bioeconomy. Coffee silver skin (CSS) and almond skin are generated in large quantities but remain underutilized despite their nutritional potential. This research aimed to use these by-products as a substitute for flour in muffins to boost total dietary fiber (TDF) and total phenolic content (TPC). Comprehensive analyses were conducted, including nutritional composition, mineral contents, TPC, antioxidant potential (DPPH, FRAP, ABTS), rheological analysis, structural characterization, and functional group identification through SEM and FTIR spectroscopy. The substitution of CSS and almond skin significantly improved the protein content (17–33%), TDF (53–113%), and TPC (16%–114%), and the 7% almond skin +5% CSS formulation showed the highest values. Rheological and SEM findings confirmed that the combination of CSS and almond skin improved the microstructure of muffins. The combination of CSS and almond skin represents promising functional ingredients for producing nutritionally enhanced bakery products.

## Highlights

• Substitution of wheat flour with coffee silver skin (CSS) and almond skin at 5–12% in muffins.
• Muffins with CSS and almond skin showed an improved nutritional profile.
• The integration of CSS and almond skin improved the texture and microstructure of muffins.
• FTIR spectra confirmed the high-intensity peaks for the integrated samples.
• The integrated samples (F5 and F6) qualify as a “source of fiber” per EU Regulation 1924/2006.

## Graphical abstract

**Figure:**
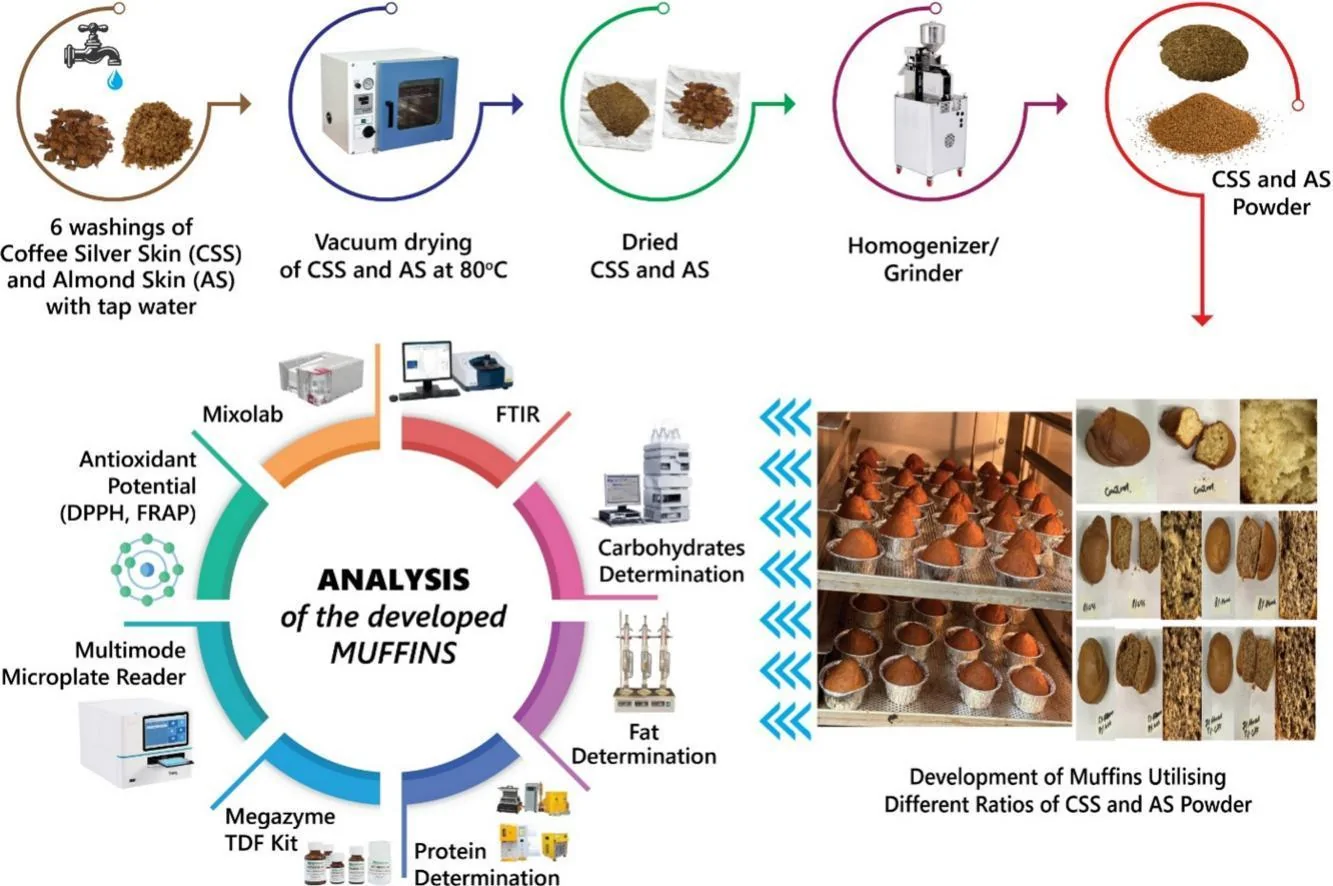

## 1 Introduction

It is estimated that the global production of food waste and by-products will increase by over 200 million tons by 2050 (Sisti et al., 2021). Agro-industrial by-products are becoming essential resources in the global initiative to mitigate food waste and push towards a sustainable bioeconomy (Valenti et al., 2023). The coffee silver skin and the almond skin are among the by-products that are produced in significant amounts but are not utilized to their full potential (Freitas et al., 2023; Nolasco et al., 2022). These by-products have been used as landfill waste and for low-value utilization as animal feed, fuel, and compost (Divyashri et al., 2026; Silva et al., 2025). The global production of coffee beans for 2023/2024 was recorded as 10 million metric tons, while the global production of almonds for the same year was 1.5 million metric tons. These by-products follow different processing conditions. CSS is produced in major coffee-consuming countries (EU countries) instead of major coffee-producing countries (Brazil, Colombia, Ethiopia) because CSS is generated during the roasting stage (Meliany et al., 2025). In 2025, the EU imported 2.9 million tonnes of coffee from non-EU countries, where 97% of the imported coffee was green coffee beans, showing Europe's role as a major player in value creation after import. Among the major coffee-consuming countries, Germany is top on the list, while Italy is 2nd and leads the way for roasted coffee production (Europe, 2026). Similarly, the USA, Spain, and Australia are among the major almond-producing countries (Alhas-Eroğlu, 2026). Almonds are traded along with the kernel, and the skin is removed during blanching and is only required if white kernels are needed for beverages or bakery products.

CSS accounts for approximately 4% of the coffee bean's weight. During roasting, the thin layer of silver skin gets separated from the bean (Nolasco et al., 2022). Similarly, almond skin is derived during the industrial processing of almonds. Almond skin is separated from the almond kernel during hot water blanching and accounts for 4–8% of total shelled almond weight (Freitas et al., 2023; Mandalari et al., 2010). These skins are an excellent source of dietary fiber, polyphenols (including flavonoids, chlorogenic acid, caffeic acid, and tannins), and other unique bioactive compounds, such as xylooligosaccharides and phytosterols. CSS additionally contains melanoidins and caffeine. The chemical composition and bioactive profile of both skins are presented in Table 1. This excellent profile of these skins makes them suitable for use as a source of dietary fiber, nutritional, and phenolic compounds in functional foods.

**Table 1:** Composition of CSS and almond skin.

Compound	CSS	Almond skin	References
Total dietary fiber	50–70%	45.10–60.25%	(Bertolino et al., 2019; Silva et al., 2025)
Fat	3%	9.50–24.20%	(Andrade et al., 2022; Silva et al., 2025)
Protein	18.9%	10.99%	(Ingegneri et al., 2023; Nolasco et al., 2022)
Melanoidins	17–23%	Absent	(Bessada et al., 2018)
Total phenolic content (mg GAE/100 g)	526.4 ± 16.3	438.6 ± 21.8	(Moreno Gracia et al., 2021; Oktay et al., 2025)
Alkaloids (caffeine, trigonelline)	27.7–34.3 mg/g for caffeine and 16.3–23.7 mg/g for trigonelline	Absent	(Castaldo et al., 2020)
Chlorogenic acid	588.9 mg/100 g	1.09 mg/100 g	(Bresciani et al., 2014; Garrido et al., 2008)
Tannin (proanthocyanidins)	309 mg/100 g (60% ethanol)	162 mg/100 g	(Bolling, 2017; Franca et al., 2024)
Quercetin	0.153–0.246 mg/100 g	0.09 mg/100 g	(Arangia et al., 2023; Nzekoue et al., 2020)
Phytosterols	984 mg/100 g β-sitosterol (771 mg/100 g)	270 mg/100 g	(Grundy et al., 2016; Nzekoue et al., 2022)
Major compounds	5-caffeoylquinic acid (199 mg/100 g), 3-caffeoylquinic acid (148 mg/100 g)	Isorhamnetin-3-O-rutinoside (41.68–74.14 mg/100 g), catechin (15.79–36.63 mg/100 g), epicatechin (9.49–26.62 mg/100 g).	(Bolling et al., 2009; Bresciani et al., 2014)

The phenolic compounds in CSS, such as chlorogenic acid, caffeic acid, and melanoidins, greatly contribute to its antioxidant potential. Similarly, almond skin was rich in flavonoids such as isorhamnetin-3-O-rutinoside, catechin, and epicatechin. The antioxidant properties of phenolic and flavonoid compounds from CSS and almond skin protect against oxidative stress and reduce the risk of chronic diseases (Garofalo et al., 2024; Regazzoni et al., 2016). The high dietary fiber content of CSS and almond skin unlocks their potential as prebiotics. Prebiotics are components of food that the body cannot digest and that selectively promote the growth and/or activity of beneficial bacteria in the gut (Valero-Cases et al., 2020). Research suggests that almonds can significantly increase the gut microbiota diversity and composition, particularly the populations of *Bifidobacterium, Roseburia*, and *Lactobacillus* species (Tahiri & Gilbert, 2025). Although studies are available on using CSS and almond skin individually, in various bakery products, they were limited to gluten-free products (addition of almond skin in gluten- and lactose-free products) (Vuono et al., 2024). The wheat matrix behaves differently, and fiber from these skins competes with gluten for available water, and this affects dough development, crumb structure, etc. CSS-based products showed a hard texture as the CSS percentage increased due to the presence of high insoluble fiber (85% in CSS) (Ateş & Elmacı, 2019; Bertolino et al., 2019; Cantele et al., 2022). This research aimed to improve the TDF and phenolic content and to evaluate the feasibility of substituting wheat flour with varying concentrations of CSS flour/powder and almond skin, individually and in combination, for muffin development. The developed muffins were stored in the freezer at −20 °C and used for further analysis. The formulated muffins were examined for microbiological analysis, proximate analysis, sensory evaluation, total phenolic content (TPC), TDF, and antioxidant capacity using DPPH, FRAP, and ABTS assays (expressed as Trolox equivalents). The combined interaction of CSS and almond skin, along with sensory, rheological, structural, and morphological characterization (through SEM), and functional group identification (through FTIR), is the novelty of our research.

## 2 Materials and methods

### 2.1 Sample collection and preparation

Raw coffee silver skin (CSS) was collected as a blend of *coffee arabica* and *coffee robusta* from Morino, Italy. Raw CSS was initially subjected to 6 manual washings with tap water (25 °C) until the wash water became transparent, then dried at 80 °C in a Binder vacuum dryer until the dryer reached a pressure below 5 millibars (mbar) (≈24 h.). The dried CSS was converted into flour/powdered form using an HMHF turbo homogenizer (high-speed blade homogenizer from PBI International, Milano, Italy). Powders were ground for 90 s using a homogenizer, sieved through a 1 mm sieve, and further used for product development. The almond skin used in this study was obtained as a by-product of the industrial almond peeling process carried out by Damiano S.p.A. Skin removal was performed by wet peeling (blanching). Almond kernels of the Marcona cultivar (*Prunus dulcis* (Mill.) D.A. Webb), originating from Spain, specifically from the Almería (Andalusia) and Alicante (Valencian Community) growing areas, were subjected to a hot-water treatment at approximately 90 °C to promote skin hydration and softening and to reduce adhesion between the skin and the kernel. Immediately after the thermal treatment, the peeling unit mechanically separated the skins from the kernels using combined compression and friction forces. The separated skin fraction was subsequently collected and dried by Damiano S.p.A. in a belt dryer at 80 °C to reduce its moisture content and ensure adequate stability during storage. We obtained approximately 5.2 kg of dried almond skins from 100 kg of shelled almonds, corresponding to a skin yield of approximately 5.2% (*w*/w), calculated as the weight of the dried skins relative to the initial weight of the almond kernels.

The dried whole almond skins were then supplied to CreoFood – Innovation and Concept Food Lab, where they were processed to obtain almond skin powder. Specifically, the dried skins were ground using a cutter and subsequently sieved through a 1 mm sieve. The fraction passing through the sieve was collected and used as almond skin powder in the subsequent experimental trials. Both the powders investigated were easily passed through a 1 mm sieve and had a particle size of 400 μm.

### 2.2 Development of muffins using different ratios of CSS and almond skin powder/flour

Different muffin formulations were prepared using various ratios of CSS and almond skin (Table 2). The rationale behind using these formulations was that a preliminary trial of 10% CSS was unacceptable due to the rubbery and hard texture (informal evaluation); therefore, 8% was fixed as the individual maximum. Neither the maximum individual concentration of CSS nor almond skin passed the Regulation (EC) No 1924/2006 for the source of fiber food (3 g/100 g) (see Results and Discussion); therefore, a combination of CSS and almond skin was used to evaluate the interaction of two different matrices. Almond skin was matched to CSS so that both by-products could be compared at equal inclusion levels. For integrated/combined formulations, the total combined substitution was set at 12%, where CSS was kept at or below 7% and almond skin provided the balance. To investigate the contribution of each by-product at constant total inclusion, the ratio between CSS and almond skin was reversed (Table 2). 3 baking batches were performed for each formulation on 3 different days, which were considered biological replicates (*n* = 3). Muffins were developed following Grasso et al., 2021, with some modifications. Italian type 00 wheat flour was used in muffin development due to its ultra-fine particle size and medium-to-low protein content (11%), suitable for cakes, pastries, and muffins. The recipe of the muffins was based on 100 g, and all the given quantities were listed as a batch of 200 g. A slightly higher egg concentration was used to develop a finer texture and better air space development, which might be affected by the addition of ingredients of interest (CSS and almond skin). Furthermore, Grasso et al. (2021) used up to 20% egg in the formulations as a modified recipe.

**Table 2:** Formulation plan for muffins using different concentrations of CSS and almond skin.

Formulation	CSS %	Almond skin %	Flour %
F_0_ (control) (0% substitution)	0	0	100
F_1_	5	0	95
F_2_	8	0	92
F_3_	0	5	95
F_4_	0	8	92
F_5_	5	7	88
F_6_	7	5	88

All other ingredients and processing time for muffins were the same. Other ingredients included 1 whole egg (43 g), 40 g of refined sugar, 2 g of baking powder, 25 g of butter, and 30 mL of milk. Muffins were baked at 175 °C for 20 min in the preheated oven, cooled to room temperature, sealed in vacuum bags, stored in the freezer at −20 °C, and then used for further analysis. Single whole frozen muffins from 3 different batches were thawed at room temperature for 2 h (covered in sealed vacuum bags) and were ground for 1 min using an HMHF turbo homogenizer (high-speed blade homogenizer from PBI International, Milano, Italy) before performing any analysis and stored in test tubes. Powdered muffin samples from 3 different test tubes were taken as replicates for individual analysis.

### 2.3 Microbiological analysis of baked muffins

The microbial safety of muffins was assessed using plate count agar (PCA) and dichloran rose bengal chloramphenicol (DRBC) for the enumeration of total aerobic mesophilic bacteria and yeast and mold detection, respectively. 10 g of sample from each muffin was aseptically weighed and homogenized in 90 mL of Ringer's solution using a stomacher. PCA plates were incubated at 30 °C ± 1, and DRBC plates were incubated at 28 °C ± 1 for 72 h.

### 2.4 Nutritional analysis

The nutritional composition of frozen muffins was evaluated. Total protein, total dietary fiber, total fat, and carbohydrate concentrations were determined, along with energy (Kcal/100 g). Energy values were calculated using the following conversion factors according to Regulation (EU) No 1169/2011 ANNEX XIV (European, and Council of the European, U, 2011).

$$\mathrm{Energy}\frac{\mathrm{Kcal}}{100\;\left(g\right)}=\left(4*\mathrm{carbohydrates}\right)+\left(4*\mathrm{protein}\right)+\left(9*\mathrm{fat}\right)+\left(2*\mathrm{fibre}\right)$$

#### 2.4.1 Total protein

The protein fraction in the developed muffins was determined using the Kjeldahl procedure, which measures nitrogen content. 0.1 g of the ground sample was placed in a digestion tube along with 1 catalyst tablet and 7 mL of sulphuric acid. After digestion, the digestate was thoroughly diluted with deionized water, and the sample tube was immersed in a boric acid solution to proceed with distillation and completion of the analysis. Finally, samples were titrated with a standardized HCl solution.

#### 2.4.2 Total fat

The Soxhlet apparatus was used to determine the total fat in samples, following the official method ISTISAN report 96/34 (Baldini et al., 1996). 5 g of powdered muffin was placed in the cellulose extraction thimble. The thimble was placed in the Soxhlet extractor along with petroleum ether for 3 h. The solvent was removed using a rotary evaporator, and the residue was dried at 100 °C. It was then cooled in a desiccator and weighed.

#### 2.4.3 Carbohydrates

Carbohydrate content in muffins was calculated through the difference method following the (100- (ash + fiber + fat + protein + moisture)) equation, while sugars were determined through the Fehling method.

### 2.5 Determination of TPC

Total phenolic content (TPC) was determined using a method described by Kupina et al. (2018), with some modifications. 1 g of powdered muffin sample was used in 10 mL of 70% ethanol for TPC extraction by sonication at 40 kHz for 60 min. Later, centrifugation and ultracentrifugation were performed at 6000 rpm for 15 min and 15,000 rpm for 5 min, respectively. 50 μl of an aliquot or standard was used in each well of a 24-well plate. Spark plate reader (TECAN, Austria, GmbH, Untersbergstr) was used for measuring the absorbance. Absorbance was measured at 765 nm after 1 h at 37 °C. TPC values were expressed as milligram gallic acid equivalents (GAE) per gram of sample (mg GAE/g).

### 2.6 Quantification of TDF

TDF was quantified using the Megazyme Integrated Total Dietary Fiber Assay kit, which aligns with the CODEX Alimentarius definition of dietary fiber and has been validated as the only AOAC method 2017.16 in 2018 (Martuscelli et al., 2021). The analytical procedure involved enzymatic digestion with heat-stable α-amylase, protease, and amyloglucosidase to effectively remove starch and protein components. After enzymatic hydrolysis, the sample underwent ethanol precipitation, followed by filtration and drying of the residue. TDF content was determined gravimetrically by subtracting the residual protein and ash from the final dried mass.

### 2.7 Total antioxidant activity of muffins

The total antioxidant activity of muffins was determined using the 1,1-diphenyl-2-picrylhydrazyl (DPPH), ferric reducing antioxidant power (FRAP), and 2,2-azino-bis (3-ethylbenzothiazoline-6-sulfonic acid) (ABTS) assays and expressed as Trolox equivalents (TEAC).

#### 2.7.1 DPPH

The DPPH assay was used to assess the radical-scavenging activity of muffins. DPPH was measured using a method described in (Kedare & Singh, 2011), with some modifications. A DPPH working solution was prepared by mixing DPPH solution (0.00789 g DPPH in 100 mL 70% ethanol) at 10:9 with acetate buffer (50 mM, pH 5.5). Trolox stock solution (1 mM) was prepared by dissolving 0.00625 g of Trolox in 50 mL of ethanol. Only 20 μl of the sample or standard, along with 150 μl of DPPH working solution, was used in a 96-well plate. The absorbance was measured at 517 nm after 30 min at 37 °C.

#### 2.7.2 FRAP

Antioxidant activity was determined using the FRAP method (Benzie & Strain, 1996; Danet, 2021), with some modifications. An acetic acid buffer (pH 3.6) was prepared by dissolving 0.45530 g of anhydrous sodium acetate (CH_3_COONa) in 50 mL of distilled water (DW). The solution was diluted to make the final concentration of 0.3 mol/L. 2,4,6-Tripyridyl-S-Triazine (TPTZ) solution was prepared using 0.1562 g of TPTZ in 50 mL of DW. 0.17 mL of 37% concentrated hydrochloric acid was added, and the solution was diluted to a final volume of 100 mL. Similarly, a FeCl_3_ · 6H_2_O solution was prepared with 0.27003 g of FeCl_3_ · 6H_2_O in 50 mL of DW. A FRAP working solution was prepared using a 10:1:1 ratio of acetic acid buffer, TPTZ solution, and FeCl_3_ · 6H_2_O, respectively. The solution was incubated at 37 °C and used within 1–2 h. Absorbance was read at 593 nm after 15 min.

#### 2.7.3 ABTS

Antioxidant activity was determined using the ABTS assay as described in Ozgen et al. (2006), with some modifications. Initially, 2 mL of potassium persulfate solution (prepared by dissolving 0.0166 g of potassium persulfate in 25 mL of ultrapure water) was mixed with 2 mL of ABTS solution (prepared using 0.0384 g of ABTS in 10 mL of ultrapure water). This 4 mL solution was diluted with an acetate buffer solution. Absorbance was measured at 734 nm after 1 h at 37 °C.

### 2.8 Mineral analysis

Minerals and trace elements in muffins were determined using the ICP-MS method described by Nolasco et al. (2022). The detection and quantification were performed using a Thermo Scientific TM ICAPTM RQ inductively coupled plasma mass spectrometer (Q-ICP-MS) with a Burgener Mira-Mist nebulizer, a quartz cyclonic spray chamber cooled to 2.7 °C, and foam cones. All formulations were analyzed in triplicate.

### 2.9 FTIR analysis

FTIR analysis was performed using a Cary 630 FTIR (Agilent Technologies, USA). Overall, 128 spectra/scans were obtained in the 4000–652 cm^−1^ range with a resolution of 4 cm^−1^. To test repeatability, analyses were performed in triplicate, and average spectra were used. Spectra were analyzed using Agilent MicroLab software, version 5.8, which was purchased with the instrument.

### 2.10 Rheological analysis of muffins using mixolab and texture profile analyzer

The thermomechanical behavior of dough was evaluated using a Mixolab® 2 apparatus (Chopin Technologies, France) following the Chopin+ protocol in accordance with AACCI method 54–60.01 (AACCI, 2010). Flour samples (75.0 g, 14% moisture basis) were mixed with distilled water to reach a target optimum torque of 1.10 ± 0.05 Nm, with water absorption (WA %) defined as the percentage of water required to achieve this torque. Testing was conducted at a constant mixing speed of 80 rpm using a temperature profile consisting of an initial isothermal step at 30 °C for 8 min, heating to 90 °C at 4 °C/min, holding at 90 °C for 7 min, cooling to 50 °C at 4 °C/min, and a final holding step at 50 °C for 5 min. The recorded torque parameters included dough stability (min), protein weakening (C2, Nm), starch gelatinization (C3, Nm), hot gel stability (C4, Nm), and retrogradation (C5, Nm). The torque difference (C5 - C4) was calculated to evaluate the extent of starch retrogradation. A stable microsystem TA-XT texture analyzer was used for firmness (N) and springiness (%). Cubes measuring 25 × 25 × 25 mm were cut from the center of each muffin and subjected to a compression test using a P/36R cylindrical probe. The probe operated at a test speed of 1.7 mm/s and applied a compressive force until the sample was deformed to 25% of its original height. Crumb firmness was expressed as the maximum force (N) required to achieve this level of compression.

### 2.11 Scanning electron microscopy (SEM)

The internal morphology of muffins was investigated using SEM (Fei Nova 450 Nano SEM, Netherlands). SEM was performed at LUMS Pakistan. Crumbs were manually sectioned to dimensions not exceeding 1 cm in each direction, and these were properly mounted on the glass slides with silver paint. Everhart-Thornley detector (ETD) with secondary electron mode was used to capture all micrographs with an accelerating voltage of 5.0 kV. All formulations were investigated at magnifications of 2500×, 1000×, 500×, and 250×, with scale bars of 20, 50, 100, and 200 μm, respectively.

### 2.12 Sensory analysis

The sensory analysis of freshly baked muffins developed with CSS and almond skin was done through a 9-point hedonic scale (Tukassar et al., 2023). The sensory analysis was performed at the National Institute of Food Science and Technology (NIFSAT), University of Agriculture, Faisalabad (UAF). The sensory evaluation was conducted by 22 semi-trained panelists. The panel for sensory evaluation comprised faculty members and postgraduate students of the NIFSAT having a doctoral degree or master's in food technology. The age ranged between 25 and 42 years, including individuals of both genders without any bias. All the panelists were informed to their consent to evaluate the product. The samples were 3-digit coded and ordered randomly to avoid any bias and artifacts associated with the sample's order of presentation. The sensory evaluation was carried out at room temperature during daytime, with ample light and an appropriate sample size for the evaluation, and the product was scored based on sensory attributes such as appearance, texture, taste, aroma, chewiness, and overall acceptability (Sharif et al., 2017). The present study was conducted in accordance with the principles established by the Declaration of Helsinki. The sensory protocol was approved by the Institutional Bioethics Committee of the University of Agriculture, Faisalabad (D. no: 184).

### 2.13 Statistical analysis

Three replications of each sample were used for analysis except for sensory (*n* = 22), and the results are presented as the mean ± standard deviation (SD). IBM SPSS® software (Version 30.0) was used to run a one-way analysis of variance (ANOVA), followed by a Tukey post hoc test to compare the impact across different formulations. The assumptions of normality and homogeneity of variance were checked using Shapiro-Wilk (for sensory) and Levene's test (α = 0.05). The RCBD within ANOVA was used as the statistical design to assess the significance (*p* < 0.05) of the various sensory parameters of the developed product, with panelists serving as blocks.

## 3 Results and discussion

### 3.1 Nutritional composition, macro and micro mineral contents and microbial analysis of baked muffins

The addition of CSS and almond skin in muffins significantly (*p* < 0.05) improved the nutritional composition, indicating that the addition of 5% and 8% CSS increased protein content up to 17.6%, while the addition of 5% and 8% almond skin increased protein content up to 20.59% and 23.53%, respectively, compared to the control sample. The protein content of the F1 and F2 formulations was the same, indicating that a 3% increase in CSS did not affect the protein content in muffins. The integrated samples showed increases of 33.82% in F5 and 23.53% in F6 formulations compared with the control. However, no significant difference (p ˃ 0.05) was observed between the CSS and almond skin formulations. Both CSS and almond skin have a varied range of protein content depending on the origin and type (Nolasco et al., 2022; Silva et al., 2025). According to our findings, the protein content in muffins was primarily contributed by almond skin. The addition of CSS and almond skin significantly reduced the carbohydrate and fat content in muffins, and these results are supported by previous literature indicating lower fat content (3%) in CSS compared with almond skin (9.50%) (Nolasco et al., 2022; Silva et al., 2025). Our results suggest that type 00 wheat flour has a higher carbohydrate content than CSS and almond skin; therefore, replacing both skins with wheat flour reduces the overall carbohydrate content.

TDF content was significantly improved by the addition of CSS and almond skin, by 53% in F1, 66.66% in F2, 80% in F3, 93% in F4, 113% in F5, and 106% in F6 formulations, as shown in Table 3.1. The main aim of the research was to enhance the TDF content of muffins, and the current results clearly confirmed an increase in TDF. TDF in integrated samples (F5 and F6) met the limit of EU Regulation 1924/2006, which states that if the product contains at least 3 g of TDF per 100 g, it may be claimed as a source of fiber (EFSA Panel on Dietetic Products, N., and Allergies, 2010). CSS contained 50–70% TDF, while almond skin ranged from 45.10 to 60.25% (Bertolino et al., 2019; Silva et al., 2025). Although both CSS and almond skin significantly improved TDF content compared with the control, the results showed that formulations containing almond skin had significantly higher TDF values than the corresponding CSS-based formulations at the same substitution level (2.7% vs 2.3% at 5% substitution and 2.9% vs 2.5% at 8% substitution) (Table 3.1). However, no significant difference was observed between F5 and F6. The nutritional values calculated in this research refer to baked muffins.

**Table 3.1:** Nutritional composition (mean ± SD) of baked muffins using different concentrations of CSS and almond skin. F0 (control-0% substitution), F1 (5% CSS), F2 (8% CSS), F3 (5% Almond skin), F4 (8% almond skin), F5 (5% CSS + 7% almond skin), and F6 (7% CSS + 5% almond skin). Differences between samples were evaluated by one-way ANOVA followed by Tukey's test. Results followed by different letters in each column were significantly different (*p* < 0.05).

Sample	Protein%	Carbohydrates%	Of which sugars	Fats%	TDF%	Energy (Kcal/100 g)
F_0_	6.8 ± 0.13^a^	55.1 ± 0.15^e^	32.4 ± 0.11^e^	12.4 ± 0.17^d^	1.5 ± 0.07^a^	362 ± 5.57^c^
F_1_	8.0 ± 0.39^b^	50.4 ± 0.10^d^	15 ± 0.09^a^	8.0 ± 0.08^a^	2.3 ± 0.16^b^	310 ± 4.58^a^
F_2_	8.0 ± 0.34^b^	50.4 ± 0.05^d^	20 ± 0.12^d^	7.8 ± 0.09^a^	2.5 ± 0.10^bc^	309 ± 6.24^a^
F_3_	8.2 ± 0.23^b^	50.6 ± 0.07^d^	19 ± 0.11^c^	8.0 ± 0.10^a^	2.7 ± 0.11^cd^	313 ± 6.56^a^
F_4_	8.4 ± 0.22^b^	49.8 ± 0.12^c^	19 ± 0.10^c^	8.4 ± 0.14^b^	2.9 ± 0.08^de^	314 ± 7.55^a^
F_5_	9.1 ± 0.15^c^	48.8 ± 0.11^b^	19.2 ± 0.06^c^	9.3 ± 0.12^c^	3.2 ± 0.08^f^	322 ± 7.00^b^
F_6_	8.4 ± 0.18^bc^	46.8 ± 0.24^a^	18.8 ± 0.13^b^	9.2 ± 0.09^c^	3.1 ± 0.11^ef^	310 ± 6.24^a^

Similarly, the incorporation of CSS and almond skin significantly affected the macro- and micromineral composition of muffins, with clear differences among formulations depending on the type and amount of the byproduct added, as shown in Table 3.2. Among macro-minerals, Ca, Na, K, and Mg were reported in muffins. Among macro-minerals, the results suggested a 79% increase in calcium concentration due to the substitution of 5% CSS, while a 98% increase in Ca concentration was observed due to the substitution of 8% CSS. The substitution of almond skin also significantly impacted Ca concentration, with an almost 53% increase compared to the control formulation. Our results for Ca confirmed that CSS was a major contributor to the Ca in muffins. Similarly, Mg was also reported to be high in CSS-based muffins, with the highest values in integrated samples, i.e., F6 (92.23%), followed by F5 (88.16%), F2 (84.71%), F1 (64.61%), F4 (51.17%), and F3 (31.93%) as compared to the control. These results are consistent with those reported by Costa et al. (2018), with CSS containing 0.584 g/100 g Ca, 2.002 g/100 g Mg, and 4.977 g/100 g K. So, replacing wheat flour with CSS improves Ca by 0.5–1 g/100 g, Mg by 2 g/100 g, and K by 5 g/100 g. According to the EU nutrient reference value (NRV) of Regulation 1169/2011, Annex XIII, our F6 formulation (40 g muffin) gives 2.5% NRV for Ca, 1.7% for Mg, and 1.6% NRV for K. To compare the mineral content such as Ca, Mg, and K with other bakery products such as muffins substituted with 15% of sunflower flour, our substituted muffins showed lower Ca (505.3 ˂780 mg/kg), Mg (155.9 ˂680 mg/kg), and K (812.5 ˂2030 mg/kg) content as compared to 15% sunflower flour substituted muffin, however CSS and almond skin improved the mineral content as compared to the control (Grasso et al., 2021). The low mineral content in our muffins may be due to the low ash content in type 00 flour (Mystkowska et al., 2024). Al and Fe were highest in CSS-based formulations, particularly F2 (14.37 and 19.21 mg/kg) and F6 (13.53 and 16.89 mg/kg), respectively. Raw CSS contains 215 mg/kg of Al and 349 mg/kg of Fe, confirming that CSS is the primary contributor of Al and Fe in formulations (Gottstein et al., 2021; Nolasco et al., 2022). Cu also followed a similar pattern as Al and Fe, with CSS-based formulations (F2, F6) showing the highest concentration. Mohammed et al. (2023) reported that the almond skin contains 148 mg/kg of zinc, and the highest amount of Zn was observed in the F4 (6.31 mg/kg) formulation in the current study, which contains a higher portion (%) of almond skin. This indicates that almond skin contributes more Zn than CSS. However, CSS-based formulations still maintain moderate Zn concentration (4.45–5.01 mg/kg), reflecting the availability of Zn from both by-products. Mn values were highest in the integrated formulations, with F5 and F6 showing the highest values (3.27 and 3.31 mg/kg), respectively. This indicates that combining CSS with almond skin improves Mn availability. Trace elements such as V, Cr, Co, and Ni showed a modest but consistent increase in CSS-based formulations, while As, Se, Mo, and Cd remained statistically the same across all formulations, indicating that neither CSS nor almond skin significantly influenced their levels in the muffins. These results suggest that the base muffin matrix could be a dominant source of these minerals. Overall, formulations with CSS showed higher mineral concentrations than formulations with almond skin. Heavy metals such as As and Cd remained statistically unchanged in all formulations. Cd values were observed at 0.06 mg/kg, which was below the limit for cereals (0.10 mg/kg) under Regulation (EU) 2023/915 (European Commission, E, 2023). Total As was measured instead of inorganic As, and there was no maximum level established. Cereal limits were used as a reference because there were no established values for bakery products.

**Table 3.2:** Minerals (Macro & micro) and trace elements (mean ± SD) Concentration (mg/Kg) in CSS and Almond skin-based muffins. F0 (control), F1 (5% CSS), F2 (8% CSS), F3 (5% Almond skin), F4 (8% almond skin), F5 (5% CSS + 7% almond skin), and F6 (7% CSS + 5% almond skin). Differences between samples were evaluated by one-way ANOVA followed by Tukey's test. Results followed by different letters in each row were significantly different at (*p* < 0.05).

bel	F_0_	F_1_	F_2_	F_3_	F_4_	F_5_	F_6_
Ca	239.2 ± 0.27^a^	429 ± 0.34^d^	475.6 ± 0.22^e^	300 ± 0.49^b^	366.4 ± 0.42^c^	434.2 ± 0.51^d^	505.3 ± 0.31^f^
Na	4.93 ± 0.08^e^	2.11 ± 0.02^b^	1.12 ± 0.03^a^	2.71 ± 0.02^c^	2.30 ± 0.03^b^	4.46 ± 0.06^d^	4.78 ± 0.09^d^
Mg	81.1 ± 0.36^a^	133.5 ± 0.23^d^	149.8 ± 0.35^e^	107 ± 0.31^b^	122.6 ± 0.41^c^	152.6 ± 0.44^f^	155.9 ± 0.46^f^
K	635.9 ± 1.67^a^	810.9 ± 1.12^d^	788.5 ± 1.19^c^	748 ± 2.11^b^	804.4 ± 2.23^d^	742.4 ± 2.19^b^	812.5 ± 2.11^ed^
Al	2.32 ± 0.05^a^	10.71 ± 0.07^d^	14.37 ± 0.07^f^	2.49 ± 0.06^b^	2.20 ± 0.07^a^	9.01 ± 0.08^c^	13.53 ± 0.08^e^
Fe	7.14 ± 0.06^a^	15.52 ± 0.08^e^	19.21 ± 0.09^g^	9.30 ± 0.07^b^	10.63 ± 0.07^c^	15.15 ± 0.05^d^	16.89 ± 0.08^f^
Zn	3.49 ± 0.07^a^	5.01 ± 0.05^c^	4.45 ± 0.04^b^	5.29 ± 0.04^d^	6.31 ± 0.05^f^	5.86 ± 0.05^e^	5.78 ± 0.04^e^
Mn	1.36 ± 0.04^a^	2.52 ± 0.07^b^	2.71 ± 0.05^c^	2.41 ± 0.06^b^	3.18 ± 0.06^d^	3.27 ± 0.05^d^	3.31 ± 0.07^d^
Cu	0.75 ± 0.01^a^	1.81 ± 0.03^c^	2.12 ± 0.04^e^	1.05 ± 0.04^b^	1.12 ± 0.04^b^	1.97 ± 0.02^d^	2.24 ± 0.06^f^
V	0.08 ± 0.01^ab^	0.10 ± 0.01^cd^	0.11 ± 0.01^d^	0.08 ± 0.01^a^	0.09 ± 0.01^abc^	0.09 ± 0.01^abcd^	0.10 ± 0.01^bcd^
Cr	0.12 ± 0.01^a^	0.17 ± 0.01^b^	0.17 ± 0.01^b^	0.16 ± 0.01^b^	0.17 ± 0.01^b^	0.22 ± 0.01^c^	0.22 ± 0.01^c^
Co	0.08 ± 0.01^a^	0.08 ± 0.01^a^	0.09 ± 0.01^ab^	0.08 ± 0.01^a^	0.09 ± 0.01^ab^	0.09 ± 0.01^a^	0.10 ± 0.01^b^
Ni	0.14 ± 0.01^a^	0.19 ± 0.04^ab^	0.20 ± 0.04^b^	0.17 ± 0.01^ab^	0.18 ± 0.01^ab^	0.19 ± 0.01^ab^	0.21 ± 0.01^b^
As	0.03 ± 0.01^a^	0.03 ± 0.01^a^	0.03 ± 0.01^a^	0.03 ± 0.01^a^	0.04 ± 0.01^a^	0.03 ± 0.01^a^	0.03 ± 0.01^a^
Se	0.03 ± 0.01^a^	0.04 ± 0.01^a^	0.04 ± 0.01^a^	0.03 ± 0.01^a^	0.04 ± 0.01^a^	0.04 ± 0.01^a^	0.04 ± 0.01^a^
Mo	0.06 ± 0.01^b^	0.06 ± 0.01^b^	0.05 ± 0.01^a^	0.06 ± 0.01^b^	0.06 ± 0.01^b^	0.06 ± 0.01^b^	0.06 ± 0.01^b^
Cd	0.06 ± 0.01^a^	0.06 ± 0.01^a^	0.06 ± 0.01^a^	0.06 ± 0.01^a^	0.06 ± 0.01a	0.06 ± 0.01^a^	0.06 ± 0.01^a^

The microbial safety of baked muffins was evaluated using PCA and DRBC, but no microbial load was detected in baked muffins. The possible reason could be that both the skins were already dried at 80 °C and were free from any microbial load, and the batter underwent 175 °C during baking.

### 3.2 TPC and antioxidant potential of CSS and almond skin-based muffins

The mean ± SD for TPC content in muffins is presented in Table 3.3, demonstrating that the substitution of CSS in muffins differed significantly (*p* < 0.05) from almond-based formulations, combined formulations, and the control; however, a non-significant difference was observed between (F2 and F3) and (F4 and F6) formulations. The results showed an improvement in TPC content by 16% and 46.15% with 5% and 8% CSS, respectively, compared to the control sample. Similarly, the addition of similar levels of almond skin also significantly (p ˂ 0.05) increased the TPC content (54.1% and 84.5%, respectively). The combined impact of CSS and almond skin in muffins significantly (p ˂ 0.05) increased TPC by up to 114.93% when 5% CSS and 7% almond skin were added, compared to the control. These results suggested that CSS and almond skin are significant sources of phenolic compounds for the development of food products. Our results for TPC were supported by (Dauber et al., 2024). They developed cookies containing raw coffee silverskin powder and ultrasound-treated CSS, reporting improvements in TPC content of 53.9% and 70.9%, respectively. Similarly, skin from coffee arabica and robusta was used in vegan biscuits (Cantele et al., 2022). The developed biscuits showed a 19.51% and 39.02% increase in TPC with the addition of 6% skin from arabica and robusta, respectively. Our results for TPC improvement are consistent with those of Vuono et al. (2024), in which TPC content was increased in gluten- and lactose-free muffins enriched with 6% almond skin.

**Table 3.3:** TPC (expressed as mg of gallic acid equivalent, GAE/g) and total antioxidant capacity of baked muffins as Trolox equivalent (TEAC mg/g of sample) using DPPH, FRAP, and ABTS Assays. F0 (control), F1 (5% CSS), F2 (8% CSS), F3 (5% Almond skin), F4 (8% almond skin), F5 (5% CSS + 7% almond skin), and F6 (7% CSS + 5% almond skin). Differences between samples were evaluated by one-way ANOVA followed by Tukey's test. Results followed by different letters in each column are significantly different at (*p* < 0.05)

Sample	TPC	DPPH	FRAP	ABTS
F_0_	0.743 ± 0.043^e^	3.406 ± 0.135^a^	0.960 ± 0.113^a^	13.356 ± 0.125^a^
F_1_	0.862 ± 0.068^d^	5.217 ± 0.105^b^	1.396 ± 0.147^b^	14.587 ± 0.141^b^
F_2_	1.086 ± 0.136^c^	7.956 ± 0.176^d^	1.963 ± 0.147^cd^	15.098 ± 0.062^c^
F_3_	1.145 ± 0.098^c^	6.666 ± 0.487^c^	1.797 ± 0.140^c^	15.423 ± 0.133^d^
F_4_	1.371 ± 0.097^b^	8.913 ± 0.192^e^	2.045 ± 0.114^cd^	15.667 ± 0.069^de^
F_5_	1.597 ± 0.331^a^	12.782 ± 0.321^g^	2.862 ± 0.173^e^	15.917 ± 0.021^e^
F_6_	1.411 ± 0.238^b^	10.783 ± 0.194^f^	2.193 ± 0.097^d^	15.812 ± 0.048^e^

The antioxidant potential of muffins was assessed using various biochemical assays. The substitution of 5% and 8% CSS significantly (*p* < 0.05) improved the antioxidant potential in baked muffins (9% and 13%, respectively), compared to the control, in the ABTS assay. Similarly, the substitution of 5% and 8% almond skin also significantly (*p* < 0.05) improved the antioxidant potential in baked muffins by 15% and 17.30%, respectively. In the combined formulation (F5), the antioxidant potential increased by 19% compared with the control. There was no significant difference between F4, F5, and F6. During the FRAP assay, substitution of CSS and almond skin also significantly (*p* < 0.05) improved the antioxidant potential by 86.56%, 113.96%, 45.31%, and 104.48% with substitution of 5% almond skin, 8% almond skin, 5% CSS, and 8% CSS, respectively. The maximum antioxidant potential was observed in F5, with a 198% increase compared to the control. Antioxidant potential was also assessed using the DPPH assay, and the substitution of CSS and almond skin significantly (*p* < 0.05) improved it in CSS- and almond skin-based muffins. F5 showed a 275% increase, while F6 showed a 216% increase in antioxidant potential compared to the control. TEAC was reported to be 80.97 (mg TE/g) in CSS extract, using 50% ethanol/H_2_O solution for 60 min (Damato et al., 2026). Similarly, 10% almond skin was used to develop waffles. The antioxidant activity ranged from 0.44 to 1.33 mmol ET/g for ABTS and 0.164–0.791 mmol ET/g for FRAP (Oliveira et al., 2023). The current findings suggest that increasing the CSS or almond skin concentration from 5% to 8% significantly (*p* < 0.05) improved antioxidant potential. Thus, almond skin-based muffins must exhibit greater antioxidant potential than CSS-based muffins. The integrated samples, containing the highest amounts of both CSS and almond skin, exhibited the highest antioxidant potential among all formulations.

### 3.3 Fourier transform infrared spectroscopy (FTIR)

FTIR is a technique used to determine the chemical composition of a material by analyzing the absorption of infrared radiation by functional groups present in the material (Dana & Sonia, 2024). The combined FTIR spectra of all 7 formulations is presented in Fig. 1, with visible frequencies at 3293, 2894, 2361, 1634, 1367, and 1025. The FTIR spectra of muffin formulations were examined to identify significant functional groups by analyzing the relevant spectral peaks. FTIR spectroscopy, which shows that there are chemical groups common in muffin compositions and examines the specific regions where these bonds absorb light. Compared with the control, this spectrum provides a detailed molecular fingerprint of muffin formulations fortified with varying amounts of CSS and almond skin. It reveals key absorption bands indicating compositional differences and interactions between functional groups.

**Fig. 1:**
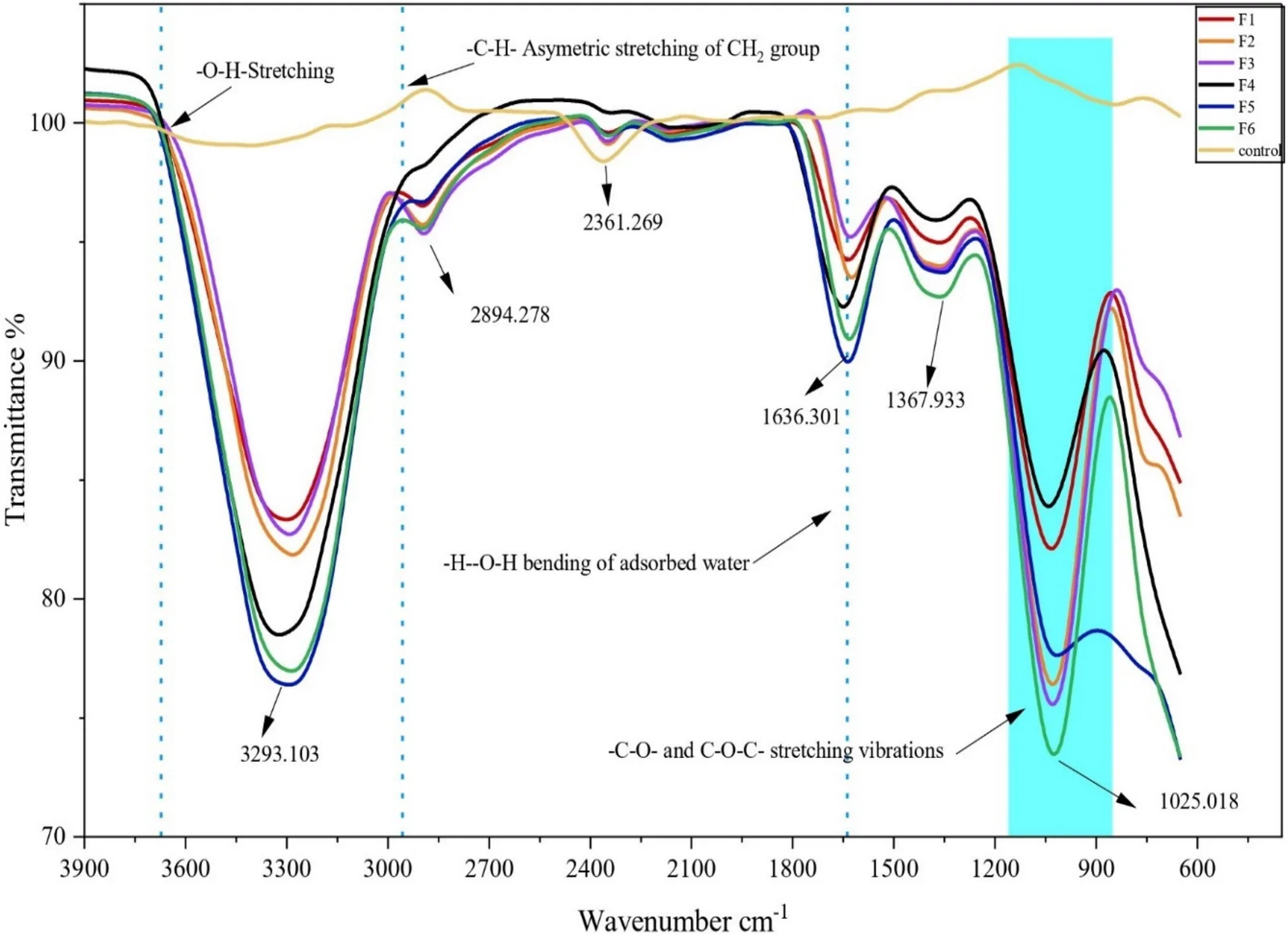
FTIR Spectra of Coffee silver skin (CSS) and almond skin-based muffins (F1-F6 and control). Spectral range from 3999 to 650 cm^−1^, and 128 scans at 4 cm^−1^ resolution. F1 (5% CSS), F2 (8% CSS), F3 (5% Almond skin), F4 (8% almond skin), F5 (5% CSS + 7% almond skin), F6 (7% CSS + 5% almond skin), and F0 (control).

CSS and almond skin are rich in both soluble and insoluble fiber. Based on the analysis of the bands, it could be determined that CSS and almond skin-based formulations displayed 6 distinctive broad absorption bands with frequencies at 3650–3000 cm^−1^ (-O-H-stretching vibration of hydroxyl group) 2950–2800 cm^−1^ (the methyl and methylene C—H stretching vibrations), which demonstrated the presence of the typical structure of polysaccharide compounds, 2410–2200 (-C-H- asymmetric stretching),1720–1500 (-H-O-H adsorbed water bending), 1400–1320 (phenolics-OH bending) and 1200–870 (-C-O and C-O-C stretching vibrations (Quintero Ruiz et al., 2021).

The peak at 2894 cm^−1^ corresponds to the asymmetric stretching vibrations of C—H bonds in aliphatic chains (CH2, CH3). These groups are typical of fats (lipids) and triglycerides that are present in the muffins through oil or other added fats (Kaur et al., 2019).

A slight increase in F3 and F4 spectra, which are enriched with AS, might be caused by some unsaturated fats that are inherent to almond skin. This observation overlaps with the vicinity of symmetric C—H modes and can be extended to oxidative stability, in which an increase in skin-derived phenolics can protect against rancidity. The peak at 1636 cm^−1^ could be due to adsorbed water bending or aromatic benzene in lignin, as observed in all formulations. At this wavelength, the O—H bending of adsorbed water co-occurs considerably with the amide I (C=O stretching) of the proteins and aromatic C=C vibrations arising from lignin in CSS or polyphenols in AS. The resulting enhanced absorption in combined formulations such as F5 and F6 may indicate protein-polyphenol complexation. Likewise, C—H bending of methyl groups, O—H in-plane bending of phenolics, and amide III are also contributing to the secondary O—H bending at 1367.933 cm^-1.^ It exhibits a greater divergence in the fingerprint region, with fortified samples, particularly F1 and F2 with CSS, having lower transmittance levels. Similar studies were also reported by Nolasco et al. (2022). The FTIR spectra of CSS were compared with those of pure cellulose. According to their findings, the strong absorption bands (1500–1750 cm^−1^) covered the aromatic ring and carbonyl vibration regions. In our formulations, the presence of cellulose, lignin, and hemicellulose is indicated; the predominant peaks were observed at 1000 cm^−1^, in the 1200–1450 cm^−1^ region, and at 3350 cm^−1^. Additionally, lignin and hemicellulose spectra typically exhibit the narrow signals seen at 2800–2900 cm^−1^ (the C—H stretching region). These results were in conjunction with our CSS-based formulations. The band between 1700 and 1600 cm^−1^ is strongly associated with caffeine and chlorogenic acid. The peak at 1654 cm^−1^ is attributed to these compounds, and its intensity increases with their concentration. The broad band between 1135 and 952 cm^−1^ arises from the stretching vibration of C—O in C–O–H bonds, such as those in glycosidic bonds, and is associated with galactomannan polysaccharides (Ballesteros et al., 2014).

Lastly, C—O and C-O-C vibrations at 1025.018 cm^−1^, which indicate the presence of ether linkages in polysaccharides and glycosidic bonds in the fiber, were the most distinguishing features of formulation-to-formulation changes in the fingerprint region. The different intensities of peaks at frequency 1025.018 cm^−1^ confirm the presence of soluble and insoluble dietary fiber (hemicellulose and cellulose), which is clearer in integrated samples (Quintero Ruiz et al., 2021).

### 3.4 Rheological analysis of muffins

The firmness and springiness values obtained for the seven muffin formulations are presented in Table 3.4. The control muffin (F0) had the lowest hardness value (3.98 ± 0.62 N) of all the formulations, which is in line with its fully consolidated, continuous gelatinized starch-protein matrix, which is confirmed by SEM and offers the best crumb cell wall integrity for a soft, compressible texture. The lowest mechanical resistance to compression was observed for F0 micrographs, where smooth and continuous pore wall lamellae were observed at all magnifications due to the development of an unimpeded gluten network and complete gelatinization of starch. This finding validates the anticipated inverse correlation between crumb matrix consolidation and firmness in wheat-based baked goods (Martínez-Cervera et al., 2011; Matos et al., 2014). The mean firmness of the muffins containing 5% CSS was slightly higher than that of the control sample. Replacing 8% of CSS increased firmness by about 34% compared with the control. These systems are characterized by progressive stiffening and enrichment with insoluble dietary fiber (as shown in Mixolab data (water absorption%). The insoluble fiber fraction of the CSS is used to replace part of the wheat flour, thereby introducing particles that act as structural diluents (Ktenioudaki & Gallagher, 2012; Le & Tran, 2026). Mixing these particles disrupts the continuity of the gluten network and, in baking, competes with starch granules for available water. The substitution of wheat flour with almond skin increased the crumb firmness when 8% almond skin was added. The mean firmness was 6.77 ± 1.06 N, the highest value among the single-ingredient formulations in this study. This more vigorous reaction is probably due to the almond skin being composed of more complex substances than CSS.

**Table 3.4:** Firmness (N) and springiness (%) of muffins formulated using coffee silver skin and almond skin. Values are mean ± standard deviation (*n* = 3); CV = coefficient of variation (%). F0 (control), F1 (5% CSS), F2 (8% CSS), F3 (5% Almond skin), F4 (8% almond skin), F5 (5% CSS + 7% almond skin), and F6 (7% CSS + 5% almond skin). Differences between samples were evaluated by one-way ANOVA followed by Tukey's test. Results followed by different letters in each column are significantly different (*p* < 0.05)

Samples	Firmness (N)	CV (%)	Springiness (%)	CV (%)
F_0_	3.98 ± 0.62^a^	15.49	55.33 ± 1.39^b^	2.52
F_1_	4.26 ± 0.39^b^	9.13	56.53 ± 0.54^c^	0.95
F_2_	5.35 ± 0.40^c^	7.4	56.68 ± 0.87^c^	1.53
F_3_	5.65 ± 1.37^d^	24.31	54.72 ± 2.06^a^	3.76
F_4_	6.77 ± 1.06^f^	15.71	56.46 ± 1.64^c^	2.9
F_5_	6.32 ± 0.56^e^	8.84	57.22 ± 1.16^d^	2.02
F_6_	6.36 ± 0.77^e^	12.11	56.67 ± 1.22^c^	2.16

At 5% substitution, F3 (5.65 ± 1.37 N) was significantly firmer than F1 (4.26 ± 0.39 N; *p* < 0.05). At 8% substitution, F4 (6.77 ± 1.06 N) was the highest value of firmness among all single-ingredient formulations, much higher than F2 (5.35 ± 0.40 N) at the same CSS level. The increased firming of almond skin compared to CSS is consistent with the SEM observation of increased matrix disruption at similar substitution levels of almond skin formulations, due to the lamellar geometry of the almond skin testa cell wall fragments (offering more matrix-disrupting interfacial area per particle than CSS cylindrical fibres), the higher total phenolic content of almond skin (especially condensed tannins), which disrupts gluten network crosslinking more extensively by proanthocyanidin–protein interactions, and the additional contribution of almond skin soluble fiber (pectin-type polysaccharides) to batter water redistribution(Garrido et al., 2008; Le Bourvellec & Renard, 2012; Sang et al., 2002).

The firmness findings for the combined formulations (F5: 6.32 ± 0.56 N; F6: 6.36 ± 0.77 N), which had a total by-product substitution of 12% in both cases, showed a statistically significant difference, though lower in absolute terms (0.41 N–0.45 N) than those of F4 (6.77 N; *p* < 0.05), the firmest single-ingredient formulation at only 8% almond skin. This was an unexpected but scientifically interesting outcome, because the simultaneous presence of both fiber types (CSS and almond skin) in the matrix produces a softer crumb than the most disruptive individual skin (F4) at lower concentration. F5 and F6 contain lower concentrations of almond skin as compared to F4. These results were in accordance with those stated by (Gularte et al., 2012) where blended fiber sources (oat-Inulin combination) showed lower hardness (5.3 N) as compared with single inulin (6.1 N). The springiness values were comparable in magnitude among all formulations (54.72–57.22%) with statistically significant but quantitatively small differences between the groups. The highest springiness was obtained for F5 (57.22 ± 1.16%), while the lowest was observed for F3 (54.72 ± 2.06%).

The results of the Mixolab (Table 3.5) indicated that both by-products had a substantial effect on the dough's thermomechanical behavior. This modification impacted hydration (WA %), structural deterioration (protein weakening-C2), starch gelatinization (C3), and retrogradation(C5). Water absorption rose progressively from 60.0% in control to 65.0% in integrated formulations, with the CSS-containing formulations showing the largest increase. The increase may be owing to the strong water-binding capacity of insoluble fiber fractions, melanoidins, cellulose, and hemicellulose contained in CSS. With the addition of fiber, dough stability generally increased (F0: 9.85 min < F3: 10.35 min < F2 ≈, F4 ≈, F6: 10.70 min < F1 ≈, F1: 10.70 min < F5: 10.85 min), with F5 showing the highest stability of all formulations. Together with their surprisingly lower firmness than F4 and their highest springiness (F5), the combined formulations (F5, F6) showed the lowest retrogradation values in the dataset, indicating the emergence of a unique functional profile in the binary substitution system that cannot be predicted by simply superimposing single-ingredient effects. For the development of CSS/almond skin combined functional muffins, this mixed synergistic character, positive with regard to matrix structural integrity (SEM) and texture softness relative to maximum single-ingredient substitution, elastic recovery, and anti-staling potential, defines a nuanced optimization space where the relative proportions of the two ingredients can be adjusted to balance nutritional enrichment, structural acceptability, and shelf-life performance.

**Table 3.5:** Thermo-mechanical properties of CSS and almond skin-based dough. WA; water absorption (%), stability during mixing (min), C2; minimum torque at protein weakening, C3; starch gelatinization, C4; pasting stability, C5; retrogradation, C5-C4; setback index (Nm). F0 (control), F1 (5% CSS), F2 (8% CSS), F3 (5% Almond skin), F4 (8% almond skin), F5 (5% CSS + 7% almond skin), and F6 (7% CSS + 5% almond skin). Differences between samples were evaluated by one-way ANOVA followed by Tukey's test. Results followed by different letters in each column were significantly different (*p* < 0.05).

Sample	WA %	Stability (min)	C2	C3	C4	C5	C5–C4
F_0_	60	9.85 ± 0.07^a^	0.537 ± 0.01^a^	1.724 ± 0.04^a^	1.631 ± 0.01^a^	2.468 ± 0.06^a^	0.837 ± 0.05^a^
F_1_	62	10.70 ± 0.14^bc^	0.610 ± 0.03^cd^	1.867 ± 0.01ᵇ	1.507 ± 0.02^b^	2.222 ± 0.03^cd^	0.715 ± 0.05^b^
F_2_	64	10.55 ± 0.07^bc^	0.599 ± 0.04^bc^	1.946 ± 0.02^d^	1.484 ± 0.01^b^	2.172 ± 0.03^cde^	0.688 ± 0.01^b^
F_3_	61.5	10.35 ± 0.35^b^	0.585 ± 0.03^b^	1.718 ± 0.06^a^	1.500 ± 0.05^b^	2.325 ± 0.01^b^	0.825 ± 0.02^a^
F_4_	62.5	10.70 ± 0.01^bc^	0.613 ± 0.06^cd^	1.724 ± 0.04^a^	1.445 ± 0.02^c^	2.240 ± 0.03^c^	0.795 ± 0.05^a^
F_5_	65	10.85 ± 0.07^c^	0.633 ± 0.04^d^	1.901 ± 0.03^c^	1.386 ± 0.04^d^	2.086 ± 0.02^e^	0.700 ± 0.05^b^
F_6_	65	10.70 ± 0.14^bc^	0.621 ± 0.05^cd^	1.960 ± 0.02^d^	1.403 ± 0.02^cd^	2.108 ± 0.04^cd^	0.705 ± 0.05^b^

### 3.5 Scanning electron microscopy

The surface morphology and structural characterization of muffins were analyzed using scanning electron microscopy (SEM). SEM was used at magnifications of 2500×, 1000×, 500×, and 250×, with scale bars of 20, 50, 100, and 200 μm, respectively. The substitution of CSS and almond skin led to a coarser, more irregular crumb structure in muffins compared with the F0 (Fig.2). This modification in porosity was mainly due to the higher TDF content in CSS and almond skin, which restricts water availability for gluten formation, resulting in a less uniform matrix. These findings are consistent with (Gómez et al., 2010). The crumb of the control muffin at 250× had a well-developed open-cell foam network, with a wide but uniform distribution of large pores within a thick protein & starch matrix. At 1000× magnification, the gluten protein film appeared as a smooth, uniform sheet with no visible particles or gaps, which showed that the gluten network formed freely, unaffected by other hydrophilic macromolecules or insoluble fiber particles.

**Fig. 2:**
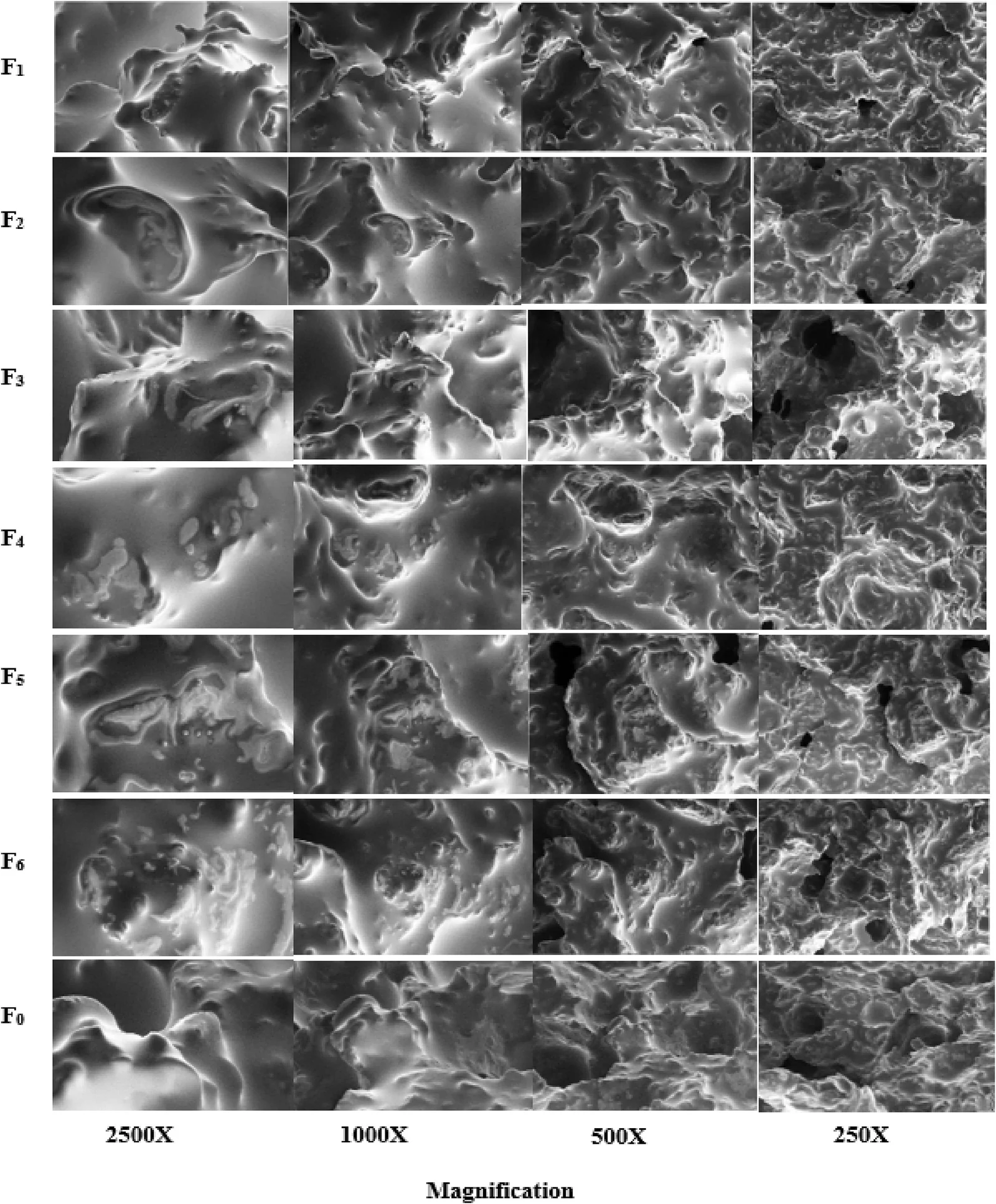
Scanning electron microscopy of muffins developed with different concentrations of Coffee Silver Skin and Almond Skin using 2500×, 1000×, 500×, and 250× magnifications. F1 (5% CSS), F2 (8% CSS), F3 (5% Almond skin), F4 (8% almond skin), F5 (5% CSS + 7% almond skin), F6 (7% CSS + 5% almond skin), and F0 (control).

The crumb structure was disrupted in a dose-dependent manner when 5% and 8% CSS were substituted for wheat flour. A significant amount of insoluble TDF is present in CSS, comprising cellulose (20–30%), hemicellulose (15–25%), and lignin (5–20%). Consequently, its viscoelastic properties, hydration, and gluten network formation are considerably different from those of refined wheat flour. Like the control, F1 exhibited a pore network but also showed localized matrix densification and increased surface roughness. The solid fraction of the matrix is locally increased in these areas due to the space-filling effect of CSS-insoluble particles that occupy intergranular spaces (Ktenioudaki & Gallagher, 2012). The structural modifications that were identified in F1 were further exacerbated in F2 as the CSS concentration increased. The image at 250× magnification revealed a vast, irregularly shaped pore. Compared to any pore that had been identified in any formulation, the pore was significantly larger and more irregular. This indicated that the gluten network's capacity to maintain gas cells in a consistent form as the batter ascended was diminished by the addition of 8% CSS. These findings are consistent with the existing understanding that substantial amounts of fiber in cake and muffin mixtures can destabilize gas cells. In those circumstances, the delicate walls were strained by fiber particles, resulting in minor ruptures and cell fusion during baking (Matos et al., 2014; Tudorica et al., 2002). Volume and height decreased due to damage to the gluten network at these higher levels. A denser, firmer-textured product resulted from the reduced gluten structure, which reduced gas retention after baking. Additionally, the muffins' mouthfeel was less smooth and grainy, due to the coarser crumb structure (Kumar et al., 2025).

At equivalent substitution levels, microstructural alterations were qualitatively distinct from those caused by CSS and were significantly more disruptive when almond skin was substituted for wheat flour. Compared with its CSS counterpart, the wall structure of F3 was more fragmented and heterogeneous in porosity at 250× magnification. This was demonstrated by the presence of pore-wall thinning and localized collapse features, which were absent at equivalent CSS substitution levels. Almond skin is exceptionally abundant in condensed polyphenols (proanthocyanidins (PA)), catechins, flavonols, and pectic polysaccharides, in addition to cellulosic and lignified cell wall material. Additionally, it has a larger interfacial area per particle due to its broad, planar, lamellar cell-wall fragment geometry as opposed to CSS's more cylindrical, fibrous geometry (Garrido et al., 2008; Mandalari et al., 2010; Sang et al., 2002). Glutamine (about 35 mol%) and proline (14 mol%) are abundant in gluten proteins and are especially suitable for complexation with PA through hydrophobic interactions and hydrogen bonds, respectively. During dough mixing, intermolecular disulfide bonds between cysteine residues play a significant role in gluten polymerization. However, as demonstrated for tannic acid, the PAs are potent antioxidants that prevent the formation of disulfide cross-links, which would weaken gluten, as shown for monomeric catechin. Therefore, although tannins have a strong antioxidant effect, their ability to enhance gluten suggests that they form extensive cross-links with gluten proteins (Girard et al., 2018). In F4, these structural changes were most pronounced in the single-ingredient formulations. The pore wall lamellae were significantly thinner and more irregular at 250× than in any other formulation. The 500× images revealed the most prominent and numerous lamellar almond skin inclusions embedded in the matrix, with the largest void formations. At 2500×, the continuous gelatinized phase of the control was most extensively disrupted, with large embedded flat-plate inclusions clearly occupying significant portions of the matrix volume and generating an almost granular, composite-like surface texture at the microscale.

At low magnification (250× and 500×), the crumb pore architecture of F5 and F6 formulations had a more spatially homogeneous pore-size distribution and a more regularly organized cell wall network. This was compared with the crumb pore architecture of F4. This demonstrated that the extreme, localized delamination indicative of excessive substitution of a single ingredient is reduced by the more uniformly distributed fiber reinforcement network formed by the simultaneous presence of almond peel and CSS in the batter.

The recognized complementarity between the two by-product fiber fractions is mechanistically consistent with the following morphological observation: CSS contributes a primarily insoluble lignocellulosic fiber with cylindrical geometry that disperses throughout the batter matrix and provides distributed structural anchoring points. Almond skin contributes both insoluble lamellar cell wall fragments and a soluble pectic fraction with significant water-holding and viscosity-modifying capacity. In addition, almond skin contributes to that pectic fraction (Ktenioudaki & Gallagher, 2012; Mandalari et al., 2010). This structural interpretation is supported by the rheological results, which showed that F5 (6.32 N) and F6 (6.36 N) were slightly softer than F4 (6.77 N; *p* < 0.05), with a difference amounting to 6.1–6.6%, despite having a significantly larger total by-product substitution of 12% compared with F4's 8%. This result shows a positive interaction between the two fiber types in reducing crumb hardness, which defies the expectation of progressive firming with increasing total fiber content. The formulation F5 exhibited the highest springiness among all formulations, at 57.22 ± 1.16%. This indicates that the crumb structure exhibited greater elastic recovery, as reported by Matos et al. (2014).

### 3.6 Sensory analysis

The sensory analysis of muffins developed using different concentrations of CSS and almond skin, including appearance, aroma, taste, texture, chewiness, and overall acceptability, is presented in Fig. 3. The addition of CSS and almond skin as a flour substitute in muffins maintained, and in some formulations improved, the sensory attributes compared to the control. However, increasing the CSS level beyond 5% led to a noticeable decline across all sensory attributes. The incorporation of up to 8% CSS in muffins reduced sensory attributes, specifically texture, taste, appearance, and overall acceptability. Our formulation F2 received the lowest sensory score from the assessors (although it was within the acceptable range of 6). The lower scores for F2 were likely associated with the high insoluble fiber content of CSS, which competes with gluten for water, disrupts gas cell formation, and contributes to denser crumb structure. Additionally, the characteristic flavor of CSS may have negatively influenced taste perception. The integrated samples (F5 and F6), which used 7% almond skin along with 5% CSS and vice versa, scored highest in all attributes except texture as compared to the control (Table S1). These results suggest a positive effect between CSS and almond skin, where the phenolics and finer particle size of almond skin may have masked the negative impact of CSS alone. All formulations received at least 6 points on the hedonic scale, with F2 receiving the lowest and F5 the highest sensory score. Sensory evaluation of F3 and F4 showed that as almond skin content increased, sensory scores decreased substantially.

**Fig. 3:**
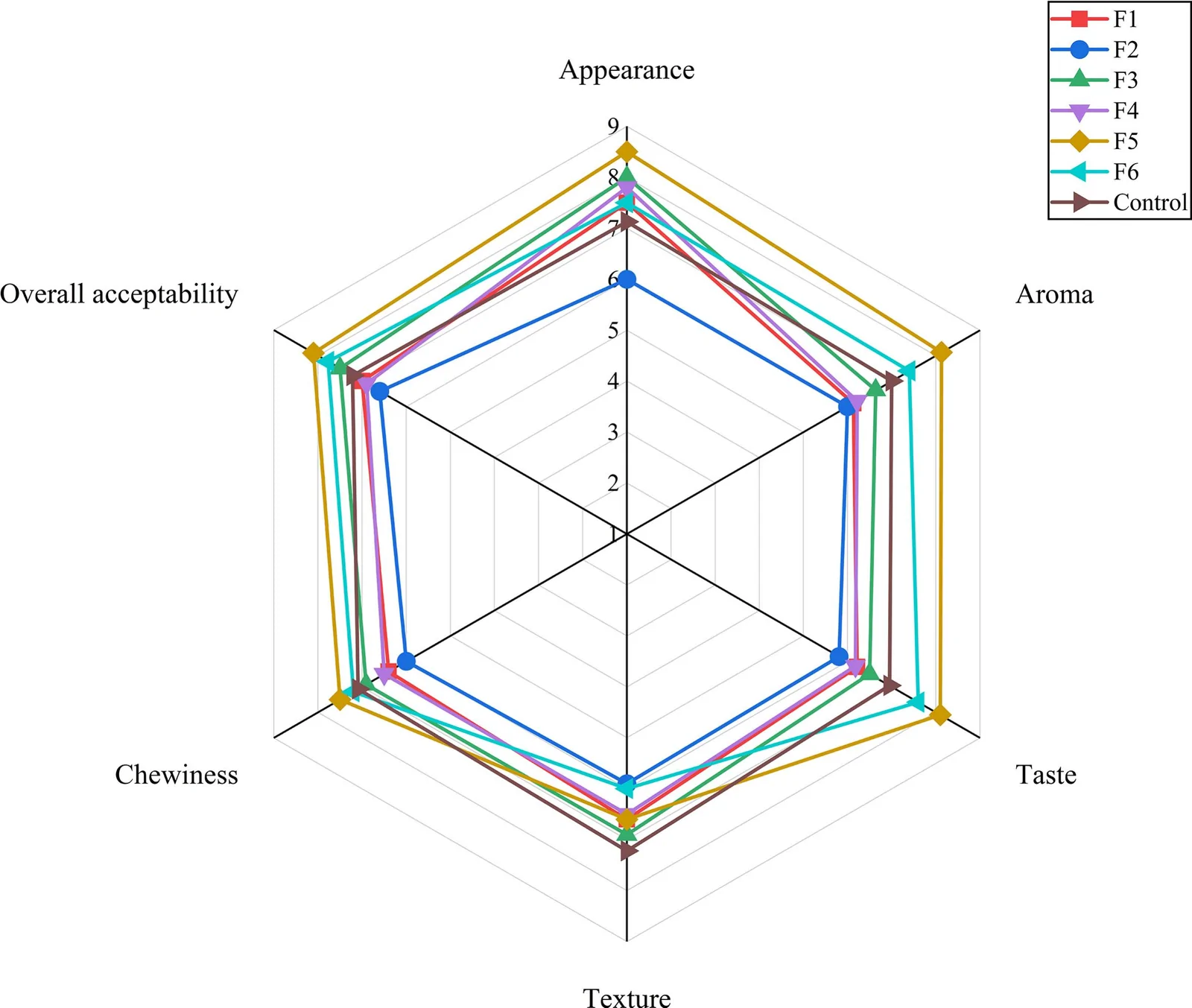
Sensory Evaluation of Muffins developed with different concentrations of Coffee Silver Skin and Almond Skin on a 9-point hedonic scale. F1 (5% CSS), F2 (8% CSS), F3 (5% Almond skin), F4 (8% almond skin), F5 (5% CSS + 7% almond skin), F6 (7% CSS + 5% almond skin), and F0 (control).

Our F5 formulation was significantly higher for taste, aroma, appearance, and overall acceptability as compared to the control. Chewiness didn't differ significantly; however, the control showed significantly higher scores for texture (Table S1). For taste, F1, F3, and F4 showed no statistical difference. Texture was the main sensory factor for the muffin, and for texture, F1, F4, and F5 showed no statistical difference. Excessive CSS (8%) likely increases water absorption and batter viscosity, leading to a harder crumb and lower taste acceptability. Although increasing the amount of both CSS and almond skin from 5% to 8% causes a reduction in sensory score, the combined effect of CSS and almond skin proved to be the best combination for the sensory properties of the muffin. This trend indicates that moderate inclusion (5–7%) of both by-products can enhance specific sensory attributes without compromising overall acceptability. CSS and almond skin are rich in insoluble dietary fiber and have high water-holding capacity, which affects the texture of the final product. The higher water-holding capacity of insoluble fiber increases batter viscosity and restricts gluten network development, which explains why the control formulation achieved higher texture scores. In conclusion, it is recommended that a combination of CSS and almond skin proved best for sensorial characteristics, thereby maximizing the utilization of waste/by-products for developing a sustainable food product. Overall, the combined use of CSS and almond skin (particularly in F5) provided the most balanced sensory profile, supporting their potential as a sustainable flour substitute in bakery products.

## 4 Conclusion

This research demonstrates that CSS and almond skin are promising functional ingredients capable of significantly improving the nutritional composition of bakery products. The inclusion of both skins in the muffin formulation led to substantial improvements in protein content, antioxidant potential, and dietary fiber. Formulations with 12% combined skins successfully met the EU Regulation 1924/2006 criteria for source-of-fiber food, confirming their potential for developing clean-labeled source-of-fiber food products. Increasing the concentration of CSS and almond skin individually above 8% adversely affects the muffin's structure, resulting in a harder, crumbly texture. However, their combined effect, up to 12%, reduced these defects, enabling greater utilization of these skins while maintaining their textural and sensorial properties. Both CSS and almond skin are rich in insoluble dietary fiber with high water-holding capacity, thereby making less water available for gluten formation and leading to the observed structural changes, which are confirmed by rheological and SEM results. Further research should focus on structural characterization using X-ray micro-computed tomography and neutron techniques to better understand the molecular interactions responsible for textural changes induced by the addition of CSS and almond skin. Further evaluation is needed to preserve blanched water (for almond skin) and water used to wash CSS, as both may contain antioxidants. The inclusion levels (up to 12%) of these by-products are limited in bakery products due to higher TDF, which affects the hydration and texture of the final product. However, optimizing green extraction techniques for polyphenol recovery and using concentrated extracts of these by-products (transforming the extract into powder via spray drying) can deliver more phenolics per gram for functional products where antioxidant capacity is the primary objective rather than fiber enrichment. Additionally, different wall materials could be added to the extract before spray drying to microencapsulate polyphenols. Microencapsulation can protect polyphenols during baking, may improve the sensory profile, and microencapsulated powder may permit higher inclusion levels. Microencapsulated extract/powder can be used for product development, and those products could be assessed through in vitro and in vivo research to better evaluate the bioavailability of phenolics and other functional compounds from baked products. The microencapsulation technique may prove beneficial for these limitations, but comes with added costs, necessitating techno-economic feasibility and life cycle assessment (LCA) of the final product.

## CRediT authorship contribution statement

**Tanveer Ahmad:** Writing – review & editing, Writing – original draft, Methodology. **Francesco Esposito:** Writing – review & editing, Supervision, Methodology. **Muhammad Arham Rauf:** Visualization, Formal analysis. **Mian Kamran Sharif:** Methodology, Data curation. **Bridget Atubukha:** Software, Funding acquisition. **Katalin Badak-Kerti:** Writing – review & editing, Visualization. **Salvatore Velotto:** Resources, Formal analysis. **Szilvia Banvolgyi:** Writing – review & editing, Validation, Formal analysis. **Teresa Cirillo:** Writing – review & editing, Supervision, Project administration, Funding acquisition.

## Declaration of competing interest

The authors declare that they have no known competing financial interests or personal relationships that could have appeared to influence the work reported in this paper.

## Data Availability

Data will be made available on request.
