# Supplementary material for: Valorization of coffee silver skin and almond skin as a source of dietary fiber, phenolics, and protein in muffins
Source: Food Chem X. 2026 Nov 4;40:104755. doi: 10.1016/j.fochx.2026.104755 (PMC13769520; doi:10.1016/j.fochx.2026.104755)
Supplement: Supplementary file 1 [file mmc1.docx]

| **Parameters** | **F1** | **F2** | **F3** | **F4** | **F5** | **F6** | **F0 (control)** |
| --- | --- | --- | --- | --- | --- | --- | --- |
| Appearance | 7.50 ± 1.57^bc^ | 6.00 ± 1.84^a^ | 8.00 ± 1.02^cd^ | 7.80 ± 1.55^c^ | 8.50 ± 1.27^d^ | 7.50 ± 1.26^bc^ | 7.13 ± 1.34^b^ |
| Taste | 6.22 ± 1.58^b^ | 5.81 ± 1.70^a^ | 6.50 ± 1.34^b^ | 6.18 ± 1.76^b^ | 8.10 ± 1.27^d^ | 7.60 ± 1.47^c^ | 6.95 ± 1.38^bc^ |
| Aroma | 6.13 ± 1.78^a^ | 6.00 ± 1.71^a^ | 6.64 ± 1.56^b^ | 6.22 ± 1.61^b^ | 8.13 ± 1.04^e^ | 7.40 ± 1.44^d^ | 7.00±1.55^bc^ |
| Texture | 6.60 ± 1.64^b^ | 5.90 ± 1.50^a^ | 6.90 ± 1.24^c^ | 6.50 ± 1.38^b^ | 6.60 ± 1.43^b^ | 6.00 ± 1.14^a^ | 7.22±1.39^d^ |
| Chewiness | 6.40 ± 1.80^a^ | 6.00 ± 1.70^a^ | 6.91 ± 1.15^b^ | 6.50 ±1.72^ab^ | 7.50 ± 1.26^cd^ | 7.20 ± 1.37^c^ | 7.09±1.75^bc^ |
| Overall acceptability | 7.00 ± 1.64^b^ | 6.60 ± 1.74^a^ | 7.50 ± 1.18^bc^ | 6.90 ± 1.84^ab^ | 8.10 ± 1.31^d^ | 7.77 ± 0.75^c^ | 7.22±1.52^b^ |

**Table S1: Sensory analysis (mean ± SD) of baked muffins.**

F0 (control), F1 (5% CSS), F2 (8% CSS), F3 (5% Almond skin), F4 (8% almond skin), F5 (5% CSS + 7% almond skin), and F6 (7% CSS + 5% almond skin). Results followed by different letters in each row were significantly different at (p < 0.05).
